# Application of transcriptomics techniques in skin cancer

**DOI:** 10.3389/fonc.2025.1688806

**Published:** 2025-11-26

**Authors:** Yuhan Ma, Fangfang Wang, Suling Xu

**Affiliations:** 1 Department of Dermatology, The First Affiliated Hospital of Ningbo University, Ningbo, Zhejiang, China; 2 Health Science Center, Ningbo University, Ningbo, Zhejiang, China

**Keywords:** skin cancer, transcriptomics, single-cell transcriptomics, spatial transcriptomics, melanoma, basal cell carcinoma, squamous cell carcinoma of the skin

## Abstract

Skin cancer is one of the most common malignant tumors worldwide, with its mortality rate showing a significant upward trend, thereby increasing the disease burden on patients and society. With the rapid development of next-generation sequencing technology, transcriptomics has revealed complex disease mechanisms by analyzing the expression patterns of differentially expressed genes, providing important tools for exploring the occurrence and development of skin cancer, studying the tumor microenvironment, investigating drug resistance mechanisms, and optimizing treatment strategies for skin cancer. This article aims to briefly summarize the development history of transcriptomics technology, review its research progress in skin cancer, and contribute to a more comprehensive understanding of the mechanisms underlying skin cancer development. It also holds promise for identifying potential therapeutic targets and providing new insights for clinical treatment of skin cancer.

## 1 Introduction

Skin cancer is one of the most common malignant tumors worldwide, with three main types (1): Malignant melanoma (MM), cutaneous squamous cell carcinoma (cSCC) and basal cell carcinoma (BCC), the latter two are classified as non-melanoma skin cancers (NMSC) (2). MM has the lowest incidence rate among these three types of skin cancer. According to statistical data, the global incidence of MM was approximately 154,000 cases in 2021 (3), constituting a mere 1% of all skin cancer cases (4). However, compared to other skin cancers, MM is highly invasive and prone to metastasis, accounting for over 75% of all skin cancer deaths (5). Most MM patients are diagnosed at an early stage, and the diagnosis is mainly stage I. Among those who undergo surgical treatment, the 5-year relative survival rate is as high as 99.5%. Patients who have not received any active treatment experience a 5-year overall survival rate of only 4.6% once distant metastasis occurs (6). BCC is the most common type of skin cancer (7), accounting for 5% of all skin cancer cases. In 2019, the incidence rate of BCC in the United States reached 525 cases per 100,000 people (8). Its global incidence rate continues to rise, with an annual growth rate of approximately 7%. While the overall mortality rate for BCC is low, the health and economic burdens on patients and their families are significantly significant (9). cSCC has the second-highest incidence rate after BCC and is the second most common NMSC. Statistics show that approximately 1 million new cases of cSCC are diagnosed globally each year, and cSCC accounts for approximately 75% of all NMSC-related deaths (10). While BCC and cSCC carry a lower risk of mortality, patients who neglect early treatment face severe consequences, including disfigurement and organ dysfunction due to tumor spread (11). In summary, these three types of skin cancer exhibit significant differences in their epidemiological characteristics. Despite the relatively low incidence rate of MM, there is a notable polarization in its survival rates. BCC is the most prevalent form of skin cancer, but it is rarely fatal. cSCC has the second-highest incidence rate but carries the highest mortality rate among NMSC. The potential threat of skin cancer to human health remains significant and should not be overlooked.

With the rapid development of next-generation sequencing technology, transcriptomics has become an essential tool in research on disease development, early diagnosis, precision treatment, and prognosis prediction, providing reliable data support for precision medicine. Transcriptomics technology is characterized by high resolution and comprehensiveness, enabling detailed analysis of all RNA molecules within cells. Transcriptomics sequencing technology is currently evolving at a rapid pace. Traditional transcriptomics technology can analyze the transcriptomic characteristics of skin cells as they progress from normal skin to malignant tumors (12), aiding in the exploration of the mechanisms underlying skin cancer development, the identification of potential diagnostic and prognostic biomarkers, and the discovery of drug resistance-related targets.

RNA sequencing (RNA-seq) technology has reached a high level of maturity. It has significantly reduced experimental costs and has increasingly standardized data analysis processes, enabling efficient comparative analysis of large-scale samples. Nevertheless, this technology still faces several significant limitations. It struggles to resolve cellular heterogeneity (13) and cannot distinguish whether differential information stems from changes in cellular composition or gene regulation (14). Additionally, the spatial positioning information inherent in transcriptome data is often lost during sample processing, which hinders the investigation of target cell functions and interactions under spatial conditions. Single-cell transcriptomics technology provides further analysis from the perspective of individual cells, constructing high-resolution cellular atlases, systematically revealing the cellular composition and differentiation trajectories of complex tissues, identifying unknown cell subpopulations and states, revealing the heterogeneity of the microenvironment in skin malignant tumors, and serving as a crucial tool for discovering precise therapeutic targets for skin cancer and developing personalized treatment plans for patients (15). Despite its demonstrated strengths, including its ability to resolve cellular heterogeneity, construct cell atlases, and infer developmental trajectories, the application of this technology is still limited by several significant factors. However, this technology exhibits deficiencies in preserving the original spatial location information of transcriptomic data during the preparation process (13). Concurrently, the presence of unique cell types may not be fully detected. Moreover, the application of single-cell transcriptomics technology is still affected by factors including substantial experimental expenses and intricate data analysis procedures. Spatial transcriptomics compensates for the loss of spatial location information of tumor cells during single-cell transcriptomics sample processing, thereby enhancing spatial resolution. By visualizing the spatial localization of different cell subtypes, we can analyze the spatial interaction networks within the tumor microenvironment (TME), providing technical support for elucidating the mechanisms of immune evasion, invasion and metastasis, and resistance to immunotherapy in skin cancer (16). Nevertheless, this technology currently faces numerous challenges. Given the nascent stage of its development, the associated technical costs are notably elevated. The analytical processes struggle to achieve a high degree of standardization. The data analysis procedures are characterized by increased complexity, and the number of genetic tests that can be conducted is constrained. The differences between these transcriptomics technologies are shown in **Table 1**. Multiple studies have reported on the application of transcriptomics in the field of skin cancer. This review will provide an overview of transcriptomics in the development and application of three major skin cancers: MM, BCC, and cSCC. It is aimed at offering new directions for research into the pathogenesis of skin malignancies and targeted therapies.

**Table 1:** Comparative analysis of transcriptomics technologies.

Transcriptomics technology	Target	Information source	Strengths	Limitations
RNA sequencing technology (RNA-seq)	Average gene expression profiles of tissue samples	Gene expression abundance	1. Technologically mature, relatively low cost. 2. Suitable for large sample comparisons.	1. Lack of cellular heterogeneity. 2. Loss of spatial location information.
Single-cell RNA sequencing technology (scRNA-seq)	Gene expression profiles in single cell samples	Gene expression abundance and cell type	1. Revealing cellular heterogeneity. 2. Building cell atlases. 3. Inferring differentiation trajectories. 4. Resolving the cellular composition of complex tissues.	1. Loss of original spatial location information. 2. Dissociation process may result in the loss of specific cells. 3. High cost. 4. Complex data analysis.
Spatial Transcriptomics (ST-seq)	Organization of gene expression profiles retaining spatial location information	Gene expression abundance, cell type and spatial location information	1. Preserve and interpret the spatial context of gene expression. 2. Locate specific gene types within tissues. 3. Study tissue microenvironments and link molecular phenotypes to tissue morphology.	1. Data analysis is more complex and requires the integration of images and multi-omics. 2. The cost of technology is extremely high. 3. The number of genes covered may be limited.

## 2 Application of transcriptomics technology in skin tumors

Transcriptomics technology is currently widely used in the study of skin-related diseases, exploring the molecular mechanisms underlying disease development, revealing the diversity and expression levels of gene transcription, and identifying disease-related signal pathways and potential therapeutic targets. In the field of dermatology, the disease with the greatest impact on patients’ survival and quality of life continues to be skin cancer. Skin cancer is one of the most destructive cancers to human health in the past decade, ranking as the fifth most common tumor (17). It is estimated that within the next few decades, it will surpass heart disease to become the leading cause of death in humans (18). The continuing advancement of transcriptomics technology has made outstanding contributions to the field of skin cancer research, providing substantial support for exploring tumor heterogeneity and resistance, potential biomarkers, the TME, the development of novel targeted therapies, and immune therapy.

## 3 MM

### 3.1 The heterogeneity and evolution of MM

**Figure 1:**
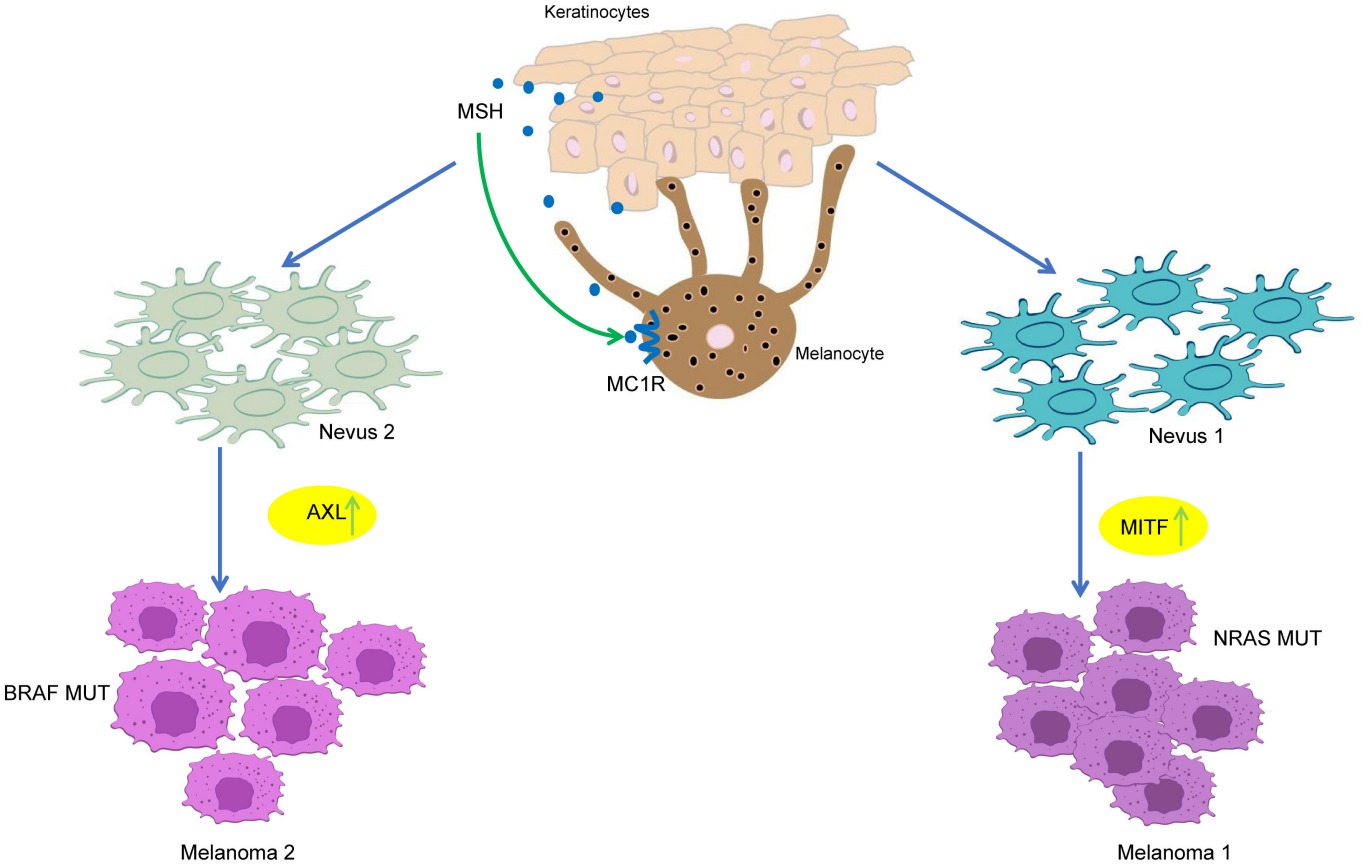
Two distinct developmental trajectories for the progression of melanocytic nevi to primary MM.

MM is the most invasive and heterogeneous type of skin cancer. It has been demonstrated by a considerable number of studies that MM is characterized by the presence of tumor heterogeneity. The observed variability in the response to immunotherapy among MM patients can be attributed to the inherent heterogeneity of the tumors. This heterogeneity leads to differing levels of sensitivity to immunotherapy, resulting in outcomes that fall short of expectations (19). Consequently, an in-depth analysis of tumor heterogeneity is not only a crucial starting point for studying the nature of MM, but also a necessary foundation for achieving precision treatment (20). Kunz et al. (21) conducted an RNA-seq analysis on primary MM samples, employing melanocytic nevi as controls. The present study revealed two distinct developmental trajectories for the progression of melanocytic nevi to primary MM and characterized the levels of treatment resistance associated with relevant mutated genes in these different trajectories. For instance, NRAS-mutated MMs demonstrate notable resistance to BRAF/MEK inhibitors, while PD-1 resistance-associated genes are highly enriched in NRAS wild-type MM. (**Figure 1**) Subhadarshini et al. integrated dynamic system modeling and transcriptomics data to validate the synergistic control of PD-L1 and IFNγ signaling by the core regulatory network in the MALME3, SK-MEL-5, and A375 MM cell lines. This study demonstrates that PD-L1 expression levels can be influenced by combining the IFNγ signal pathway with the growth and invasiveness of MM cells. This combination achieves dynamic conversion between tumor phenotypes, leading to heterogeneity and promoting immune escape (22). These findings progressively deconstruct the dynamic evolutionary landscape of MM heterogeneity, visualize the differentiation trajectory of MM through the spatial-temporal dimension, lay the foundation for the development of targeted treatment strategies, and improve the clinical response rate of immunotherapy.

### 3.2 The TME of MM

Transcriptomics is a powerful tool for analyzing TME heterogeneity and functional regulation. By conducting a comprehensive analysis of the TME, we can gain a dynamic understanding of its characteristics. Several studies have employed a combination of single-cell RNA sequencing (scRNA-seq) and spatial transcriptomics technologies (ST-seq) to analyze tumor tissue samples from patients with acral MM who have lymph node metastasis and those who have not yet developed metastasis. These studies have revealed that the TME of acral MM is significantly heterogeneous but generally highly immunosuppressed. A more pronounced reduction in invasive immune cells is observed in tissue samples from patients with lymph node metastasis (23). Parab et al. performed single-nucleus RNA sequencing (snRNA-seq) on 16,839 cell nuclei obtained from a mouse MM model with the BRAFV600E mutation and identified 11 malignant cell subpopulations. A substantial heterogeneity was identified between malignant cells and melanocyte clusters, particularly in advanced tumors, where there was a significant reduction in CD4+ and CD8+ T cell populations. Subpopulations associated with cell cycle and proliferation were suppressed (24). Gokuldass et al. applied scRNA-seq technology to sequence and analyze tumor-infiltrating lymphocytes (TILs) and autologous tumor cells, as well as 93 patients with MM or epithelial cancer. The researchers found that, despite significant differences in the TME, the overall proportion of reactive T cells was low across all tumor types. In comparison with non-melanoma tumors, MM demonstrated the most substantial CD8+ TIL reactivity (25). The study’s findings indicate that the TME of MM patients manifests a state of profound immune suppression, primarily characterized by T cell exhaustion and reduced immune cell infiltration. Mlynska and colleagues examined publicly accessible transcriptomic data from MM patients, with the objective of identifying the seven genes exhibiting the most substantial disparities in expression levels. They then constructed an immune subtype classifier and validated it using histopathological and differential gene expression analysis results from 98 surgical samples of MM patients. This approach has emerged as a new biomarker for assessing the immune microenvironment status of MM patients, providing a more precise targeted adjunctive tool for subsequent immunotherapy (26). In summary, these studies have greatly advanced our understanding of the TME and provided a critical classification tool for the development of TME-based therapeutic strategies.

### 3.3 MM immune escape and resistance mechanisms

The development of MM is accompanied by changes in related cells and regulatory mechanisms. Transcriptomics technology has the capacity to comprehensively explore the progression pattern of MM from the perspectives of the whole organism, single cells, and spatial distribution, providing a basis for its treatment. Choi et al. conducted a ST-seq analysis on different subtypes of acral MM, including amelanotic acral MM and pigmented acral MM. A comparison of amelanotic acral melanoma (AAM) with pigmented acral melanoma (PAM) revealed significant downregulation in immune regulation-related pathways, including antigen presentation and interferon signaling. These findings provide a foundation for further investigation into the differences in invasiveness and prognosis between the various subtypes of acral MM (27). Li and his team’s research demonstrates that during the transformation of moles into MM, melanocytes undergo dynamic changes through four distinct stages. In the MM stage, the expression of interferon regulatory factor 1 (IRF1) is specifically downregulated, and HLA-E, which plays a key inhibitory role on NK cells, is significantly expressed in malignant melanocytes and fibroblasts. This may be a significant factor contributing to the failure of immune surveillance and the promotion of immune escape in MM (28). The study further confirmed that the expression level of Midkine (MDK) 20 (29), which synergistically activates and regulates the NF-κB pathway in melanocytes, was significantly increased, promoting vascularization and assisting malignant tumor invasion and proliferation (28). Melanocyte-inducing transcription factor, also known as microphthalmia-associated transcription factor (MITF), plays a pivotal role in melanocyte development and melanin synthesis, and is considered a critical factor in the MM pathway (30). Ostrowski et al. employed a combination of traditional transcriptomics and scRNA-seq to ascertain that MM cell lines manifest two distinct phenotypes: proliferative (MITFhigh) or invasive (MITFlow) (31). The transition from proliferative to invasive MM is accompanied by metastasis progression. AXL, a receptor tyrosine kinase, has been found to be overexpressed in MITFlow MM cells, and its expression has been linked to tumor resistance (32). According to reports, traditional transcriptomic sequencing analysis of samples revealed that MM types with low MITF and high AXL expression are insensitive to BRAF inhibitor therapy (31). In instances of resistance to BRAF inhibitors, there is a notable upregulation in the expression of growth factor receptors (EGFR, PDGFR, EPHA 2, AXL, NGF). This offers potential as biomarkers for MITFlow or for treating resistant states, with significant clinical application prospects. The aforementioned studies have revealed several key factors related to the efficacy of immunotherapy, such as macrophage subtypes, tumor subtype classification, and B cell proportions, through in-depth analysis of the TME characteristics of MM. Tumors have been observed to circumvent the immune system by modulating a heterogeneous immune microenvironment, thereby facilitating tumor metastasis and resistance to immunotherapy.

### 3.4 Immunotherapy for MM

A study constructed co-expression modules based on RNA-seq data using weighted gene co-expression network analysis (WGCNA). Subsequent analysis of the association between these modules and MM clinical characteristics identified genes closely related to survival (CCNB2, ARHGAP30, SEMA4D). The validation of these genes was conducted through gene expression profile interaction analysis (GEPIA) and the Human Protein Atlas, indicating their potential as molecular targets for therapeutic intervention (33). Cancer-associated fibroblasts (CAFs) are a primary matrix cell type in the TME, exhibiting high heterogeneity and driving rapid tumor progression. A transcriptomic analysis of MM samples was conducted to quantify the activity of inflammatory Cancer-Associated Fibroblasts (iCAFs) in the samples. The study found that patients with high iCAF scores had longer survival times and exhibited higher sensitivity to immune checkpoint blockade (ICB) therapy (34). Neil et al. conducted an analysis of whole-transcriptome sequencing data from 328 patients with cutaneous MM. Following the classification of tumor samples according to the distribution of macrophages, lymphocytes, and monocytes, it was determined that M0 macrophage enrichment and the lymphocyte-to-monocyte ratio were independent adverse factors influencing the subsequent immunotherapy of tumors. A lower ratio of lymphocytes to monocytes in the TME was found to correspond with faster tumor progression (35). Recently, ICB therapy has achieved significant breakthroughs in the treatment of MM. This therapeutic modality involves the inhibition of immune checkpoint factors, including PD-1 and CTLA-4, to augment the body’s capacity to resist tumor development. However, the efficacy of these treatments varies from patient to patient, with approximately 55% of MM patients developing resistance to PD-1 inhibitors (36). Mallarto et al. conducted a systematic, integrated analysis of proteomics and transcriptomics data from metastatic MM patients to identify potential biomarkers associated with resistance to immune checkpoint inhibitor (ICI) therapy. This finding facilitates more precise screening of patient groups likely to be unresponsive to treatment, thus offering a novel technical pathway for predicting treatment response in metastatic MM patients (37). A team of experts conducted an unsupervised clustering analysis of mRNA transcripts from late-stage MM patients, dividing the samples into three subtypes: immune, keratin and MITFlow. The findings indicated that patients exhibiting the “immune” subtype had significantly higher survival rates after treatment in comparison to those with the keratin and MITFlow subtypes. These findings indicate the potential for immunotherapy to enhance the efficacy of treatment outcomes for late-stage stage III MM (38). Furthermore, Zhang et al. employed a combined approach of scRNA-seq and whole-transcriptome analysis of MM patient data from the Gene Expression Omnibus (GEO) dataset to investigate the correlation between the TME and immune therapy response. The researchers discovered that the proportion of B cells exhibited a significant correlation with the tumor’s response to immune therapy. To construct a predictive model for MM ICI response, machine learning methods were employed. Furthermore, it was confirmed that ITRGs can effectively predict the response of MM patients to immune therapy (39). The detection of biomarkers can also be used to identify high-risk patients for PD-1 therapy. The identification of potential genes and pathways associated with immune-related adverse events is a critical step in reducing the incidence of such events. For instance, the identification of protective genes that suppress colitis could potentially mitigate its impact on patients’ quality of life and enhance antitumor efficacy (40). Consequently, researchers developed a T-cell inflammation gene expression signature, which was derived from the expression analysis of T-cell inflammation-related genes. Its clinical predictive efficacy was successfully validated across multiple independent tumor cohorts. This signature has the capacity to identify potential responders in clinical practice, thereby enabling more precise clinical decision-making (41). These studies contribute to a better understanding of the mechanisms underlying MM’s response to ICB therapy. They also provide important theoretical foundations for developing new immunotherapy targets, identifying potential patients who may benefit from such therapies, and establishing personalized treatment plans in clinical practice. This study offers more robust preclinical evidence for exploring the clinical potential of MM immunotherapy, thereby reinforcing the feasibility and optimization directions for clinical translation pathways.

## 4 BCC

### 4.1 Heterogeneity of BCC

BCC is the most prevalent form of skin cancer. The tumor heterogeneity of BCC is intricate and consequential, playing a pivotal role in subsequent treatment strategies.

Berl et al. employed a combination of proteomics and traditional transcriptomics to elucidate the heterogeneous characteristics of different subtypes of BCC. Their findings also confirmed the presence of significant heterogeneity in samples from multiple sites within the same patient (42). In a recent study, Yerly et al. employed a multifaceted approach encompassing scRNA-seq and ST-seq to analyze invasive BCC tissue. Their analysis yielded significant insights into the composition of cell populations within the TME. The epithelial cells constituted 50.63% of the total, primarily comprising tumor cells, basal-like cells, squamous differentiated cells, and hair follicle keratinocyte subpopulations. The spatial distribution of different lesion areas was found to be heterogeneous (5). The presence of tumor heterogeneity poses challenges to the treatment of BCC, indicating that clinicians must consider factors such as tumor cell distribution and heterogeneity when devising more precise treatment plans.

### 4.2 Molecular markers of progression of BCC

In this study, researchers compared scRNA-seq data from tumors and adjacent non-tumor tissues in BCC patients. They used a method called Seurat to visualize individual BCC cells in a two-dimensional cell type map. The researchers found that KRT14+ epithelial/tumor cells clustered prominently, and malignant epithelial cells exhibited significant transcriptional heterogeneity. Among these, KRT14+ epithelial/tumor cells derived from tumor tissue significantly expressed unique BCC-related gene molecular markers, such as BCAM and EPCAM. These findings thereby identify differential molecular markers between tumors and their adjacent non-tumor tissues (43). Concurrently, the study identified a defensive response in BCC tumor cells to the inflammatory signaling pathway activated by WNT5A, manifesting as the upregulation of heat shock proteins (HSP). Consequently, HSP inhibitors may offer a promising therapeutic approach for the management of BCC (43). BCC is characterized by a low risk of mortality, with the majority of cases exhibiting an indolent course. However, invasive basal cell carcinoma (iBCC) is the most aggressive form of BCC. Employing RNA-seq technology, a team of researchers has successfully sequenced 8 iBCC samples and 21 control samples. It was found that iBCC tumor cells exhibited enhanced activation of the integrin and Wnt/β-catenin signaling pathways in comparison with non-iBCC subtypes of BCC (44). In the TME of BCC, the activation and inhibition of multiple signaling pathways play an important role in tumor growth and development (45). Morgan et al. identified that under the induction of metalloproteinases MMP3 and MMP11, CD200 undergoes exosomal domain shedding, which blocks the MARK signaling pathway, thereby weakening cell killing efficacy and causing immune escape (46). Moreover, the study predicted that CD200 could serve as a potential indicator of targeted NK cell-specific immune checkpoints (46). Huang et al. extracted 29,334 cells from iBCC and its adjacent tissues for scRNA-seq. The investigation revealed that, in comparison with normal tissues, malignant basal cells (MBCs) in iBCC exhibited reduced expression of major histocompatibility complex I (MHC-I) and a diminished capacity to interact with MHC-I signaling (HLA-CD8A). This phenomenon is known as immune escape. The study also found that as MDK signal expression levels increased, the invasion depth of iBCC increased correspondingly, confirming that MDK is an independent risk factor for predicting iBCC invasion depth (47). Moreover, Li et al. identified significantly differentially expressed genes associated with immune checkpoints using microarray data for BCC from the GEO database. Validation through multiple datasets and *in vivo*/*in vitro* experiments with VCAN knockout models demonstrated that VCAN is closely linked to BCC proliferation, migration, and invasion capabilities. These findings substantiate VCAN as a potential clinical target for BCC progression and treatment (48).

In summary, the progression of BCC is driven by multiple factors, including abnormalities in various signaling pathways and disruption of the immune microenvironment. The utilization of molecular biomarkers in monitoring these factors enables a comprehensive reflection of biological behavior, thereby providing a framework for the development of clinical treatment strategies. This, in turn, facilitates the establishment of a predictive precision medicine system.

### 4.3 The progression mechanism of BCC and immunotherapy

Ganier et al. employed scRNA-seq technology to analyze skin samples from patients with BCC and healthy individuals, thereby mapping the skin cell population landscape. RGS5+ pericytes and POSTN+ fibroblasts were found to be enriched, with the latter primarily clustered around the tumor islands in BCC. This study further confirmed the origin of malignant epithelial cells in BCC from inner and outer hair follicle cells and their high expression of PTCH1/2 and HHIP. These proteins are associated with the hedgehog signaling pathway. The result of this study indicates that these cells promote tumor cell growth and excessive proliferation (49). The abnormal activation of the hedgehog signaling pathway has been identified as a critical factor in the development of BCC. When patients with advanced or metastatic BCC do not qualify for surgery or radiotherapy, the use of SMO inhibitors offers a viable alternative treatment. SMO inhibitors function by inhibiting the activation of this pathway, resulting in the suppression of tumor growth (50). However, due to the high degree of tumor heterogeneity in advanced BCC, approximately 43% of BCC cases are resistant to SMO inhibitors such as vismodegib (51). Yao et al. found that BCC cells positive for nuclear myosin-related transcription factor (nMRTF) exhibited significant resistance to SMO inhibitors. Consequently, further scRNA-seq analysis of BCC resistant to vismodegib revealed that LYPD3, TACSTD2 (also known as TROP-2), and LY6D were significantly overexpressed in cells with high MRTF expression. The researchers confirmed that these three genes are reliable indicators of nMRTF activity and effective molecular biomarkers for assessing the prognosis of SMO inhibitor treatment (52). The basal-to-squamous transition (BST) has been observed to have a significant relationship with BCC resistance to SMO inhibitors. Through scRNA-seq and ST-seq studies, it was found that the genes PCYT2 and ETNK1, which are related to the phosphatidylethanolamine biosynthesis pathway, are upregulated during BST (53). BST manifests predominantly in the core region of tumors, exhibiting notable squamous differentiation characteristics, which may be associated with tumor invasiveness. The basal-to-inflammatory transition (BIT) is a process that occurs in the TME. In this process, IL-1 and OSM are secreted by TREM1+ myeloid cells. These cytokines activate the NF-κB signaling pathway in tumor epithelial cells. This activation promotes an inflammatory phenotype and reduces sensitivity to SMO inhibitors, such as vismodegib (54). In addition to resistance to SMO inhibitors, Pich-Bavastro et al. used scRNA-seq combined with ST-seq to discover that Activin A induces CAFs and macrophage polarization, causing the TME to become immunosuppressive, ultimately leading to stronger resistance to ICI treatment in BCC (55). The extant studies have primarily focused on the tumor heterogeneity of BCC, revealing the complexity and multifaceted nature of BCC tumor heterogeneity. Within the TME, the activation and inhibition of multiple signaling pathways provide tumor cells with a growth environment characterized by drug resistance and immune suppression. The coexistence and dynamic transformation of the two cellular states, BST and BIT, have been demonstrated to exacerbate BCC’s treatment resistance and invasive capacity. These studies provide new directional guidance and biomarker targets for immunotherapy and targeted therapy in clinical practice, offering the potential to develop more precise, personalized treatment plans for BCC patients and improve their prognosis.

## 5 cSCC

cSCC is a malignant tumor originating from keratinocytes in the epidermis or appendages. It is the second most common NMSC after BCC (56). Although more than 90% of cSCC cases are clinically benign, there is still a possibility of progression to advanced tumors and metastasis (57).

### 5.1 Transformation from normal skin to cSCC

Prolonged exposure to ultraviolet (UV) rays can trigger the transformation of normal skin into cSCC. Yan et al. utilized smart-seq2 transcriptomics technology to sequence six primary UV-induced cSCC samples. Their findings revealed that cSCC cells manifested diverse levels of chromosomal copy number variations (CNVs), with the majority of CNV levels exhibiting significant increases compared to those observed in normal control tissues. A limited number of samples exhibited lower CNV levels. This finding reveals the tumor cell heterogeneity present in cSCC and its distinct chromosomal copy number variation characteristics compared to normal tissue (58). Furthermore, it was determined that while cSCC patients exhibit a diverse array of cell populations with varying relative abundances, the predominant cell subpopulations can be categorized into five distinct classes: normal keratinocytes, fibroblasts, cSCC cells, B lymphocytes, and DC cells. The representative expression genes of each cell population demonstrate significant disparities (58). Chitsazzadeh et al. found that miR-181a was highly enriched in cSCC cells, significantly inhibiting UV-induced apoptosis, enhancing tumor invasiveness, and inducing overexpression of Epithelial-Mesenchymal Transition(EMT) -related markers SNIAL, CDH1, and SLUG through activation of the TGF-β2 signaling pathway (59). The process of UV-induced skin carcinogenesis in normal skin is accompanied by genetic mutations, the reorganization of cell subpopulations, and the activation of signaling pathways. These observations may provide precise preventive and therapeutic strategies for targeted treatment of cSCC.

### 5.2 Transformation from precancerous lesions to cSCC

Actinic keratosis (AK) is a prevalent precancerous skin lesion. Untreated AK is highly likely to progress to cSCC. Bone et al. conducted extensive transcriptomic analyses on 14 cases of AK, 44 cases of cSCC *in situ*, and 4 cases of metastatic SCC, revealing dynamic changes in the transcriptome as it progresses from a normal differentiated state to a progenitor-like state. The study revealed that long non-coding RNAs (LncRNAs) manifest differential expression across various stages of the disease and demonstrate a positive correlation with overall gene expression patterns. This observation suggests that LncRNAs can serve as a surrogate for the total transcriptome, facilitating the identification of the progression status of cSCC and the monitoring of disease progression (60). A comprehensive meta-analysis of numerous publicly accessible whole-transcriptome data sets was conducted, revealing a marked increase in the proportion of tumor-specific keratinocyte clusters (TSKs) during the progression from AK to invasive cSCC. The MMP10, PTHLH, and MMP1 genes expressed by this cell subpopulation showed a significant upward trend in expression during cSCC progression. These findings have been validated using scRNA-seq datasets, suggesting that the TSK gene signature could serve as a novel therapeutic target for the progression of AK to cSCC (12). Bowen’s disease (BD) is a cSCC *in situ* that, if left untreated, can progress to invasive SCC. During the progression of SCC, normal fibroblasts in the TME are stimulated by various tumor signals and transformed into CAFs. These CAFs assist in tumor growth, invasion, and immune evasion. A study analyzed data from BD, SCC, and healthy tissue samples, yielding over 115,000 scRNA-seq data points. These data confirmed the heterogeneity of different subtypes of CAFs in the progression of SCC. The study revealed that inflammatory CAFs (iCAFs) are predominantly present in BD, while myofibroblastic CAFs (myCAFs) are predominantly observed in cSCC. However, the presence of CAFs was not detected in AK (61). Furthermore, the study revealed that iCAF predominantly originates from pro-inflammatory fibroblasts, while myCAF primarily originates from mesenchymal fibroblasts. This finding provides a new framework for the development of targeted tumor treatments (61). The progression from precancerous lesions to cSCC is a dynamic process. The aforementioned study suggests that LncRNA can dynamically monitor changes in the transcriptome, potentially enabling early prediction and diagnosis of cSCC. Furthermore, changes in CAF subtypes within the TME have the potential to serve as prognostic indicators, offering novel research perspectives for the prevention of precancerous lesions progressing to cSCC.

### 5.3 TME and molecular mechanisms of cSCC

Specific cell subpopulations within the TME can function as pivotal regulatory nodes that govern tumor immune evasion, predicated on distinctions in molecular expression profiles. Lu et al. employed scRNA-seq to ascertain that tryptophan 2,3-dioxygenase (TDO2) expression is present in fibroblasts within cSCC, exhibiting notable heterogeneity when compared to other cell types. The study further elucidated that TDO2 expression can result in a decrease in CD8+ T cell infiltration, consequently facilitating immune evasion (62). The TME has been identified as a critical factor in the recurrence of cSCC. The TME of recurrent cSCC differs significantly from that of primary cSCC, with recurrent cSCC exhibiting T cell exclusion and a high accumulation of SPP1+ tumor-associated macrophages (TAMs). TSKs have been observed to engage in active intercellular communication with IL7R+ CAFs, resulting in the manifestation of substantial EMT characteristics (63). Ji et al. employed scRNA-seq to analyze the TME of cSCC, thereby identifying a unique set of TSKs located in the vascular niche of cSCC. TSKs have been found to express elevated levels of EMT-related molecular markers, including VIM and ITGA5. TSKs promote EMT conversion through interactions with CAFs and endothelial cells, accelerating the progression, invasion, and metastasis of cSCC and leading to poor prognosis (64). In their study, Yu et al. conducted a transcriptomic analysis on five cSCC tumor specimens, thereby revealing that the FSTL1 gene exhibited significantly elevated expression levels in cSCC. Moreover, these expression levels demonstrated a close association with the patient’s prognosis, thus aiding in the prediction of cSCC metastasis and recurrence. The team established FSTL1 gene knockout and overexpression models. Through both *in vivo* and *in vitro* validation, the team was able to elucidate that FSTL1 primarily promotes EMT in cSCC cells through the THOC7-AS1/OCT1/FSTL1 axis associated with ZEB1, thereby driving the progression, migration, and invasion of cSCC (65).

Lopez-Cerda et al. found that the transcriptome exhibits dynamic heterogeneity during cSCC progression. The study revealed that late-stage cSCC or recurrent samples exhibited activation of the epithelial-mesenchymal phenotype, indicating high invasiveness and significantly higher ITGAV expression compared to early-stage patients. This suggests that ITGAV can serve as a valuable prognostic biomarker for effectively detecting tumor recurrence (66). In the study by Yan et al., the authors investigated the role of UV-induced cSCC at the single-cell level through the use of transcriptome sequencing of tumor samples from patients undergoing anti-PD-1 therapy. Their findings revealed that S100A9 and FABP5 exhibited significant overexpression in cSCC. The validation of these findings was conducted through qRT-PCR and immunohistochemistry, which revealed the significant influence of these two genes on cell proliferation and migration through the NF-κB pathway. Consequently, S100A9 and FABP5 may offer novel targets for the development of targeted therapies for cSCC (58). The extant studies have revealed the dynamic evolution of the molecular mechanisms of cSCC and potential regulatory targets, providing important evidence for a deeper understanding of the biological behavior of cSCC and laying the foundation for targeted treatment strategies.

### 5.4 Immunotherapy for cSCC

The changes in the TME are closely associated with anti-PD-1 therapy, as evidenced by the use of cemiplimab treatment. Esposito et al. conducted gene enrichment analysis on SCC patients after cemiplimab treatment and found that the expression of genes in the IL-2/STAT5 pathway significantly increased, while the expression of interferon-related genes decreased markedly. In this study, tumor specimens and corresponding peripheral blood specimens from patients were analyzed. It was found that in patients with a favorable response to cemiplimab treatment, the abundance of CD8+ T cells and B cells in the immune microenvironment was significantly increased, while these two cell types were less infiltrated in patients with poor treatment outcomes (67). A study collected and analyzed transcriptomic data from patients with cSCC treated with cemiplimab and found that the CCL-20 and CXCL-8 (IL8) genes were significantly upregulated in patients resistant to PD-1 therapy. This upregulation promoted the aggregation of immune-related regulatory T lymphocytes (Tregs), induced their migration, led to tumor escape, and caused patients to develop resistance to cemiplimab (68). Esposito et al. monitored the levels of PD1+ Tregs in peripheral blood of cSCC patients during each treatment cycle. In the initial cycle, patients exhibiting unfavorable treatment outcomes exhibited a typical decrease in PD1+ Treg levels. However, in the third phase, patients with significantly improved treatment outcomes exhibited the opposite result. This finding supports the conclusion that the abundance of PD1+ Tregs can be used to predict the efficacy of cemiplimab, thereby enabling more precise, personalized treatment for cSCC patients (67). Subsequent studies revealed that the expression of IL-1β and IL-8 factors was reduced in responders, providing substantial evidence for their utilization as emerging biomarkers for evaluating the efficacy of anti-PD-1 therapy in cSCC patients (67).

A growing number of studies have begun to focus on the progression process of normal skin and precancerous lesions transforming into cSCC, emphasizing the important role of the TME in tumor development. By studying the TME, researchers can identify the heterogeneity of SCC, potential molecular markers, and mechanisms of resistance to immunotherapy. This provides a new theoretical basis for developing more precise, personalized immunotherapy regimens.

## 6 Conclusion

A large number of transcriptomics studies has been dedicated to the analysis of MM, cSCC, and BCC. The continuous development of transcriptomics technology has driven the advancement of research on these malignant tumors. (**Table 2**) In the domain of skin cancer research, RNA-seq technology has established itself as a cornerstone of traditional transcriptomics, playing a pivotal role in the systematic analysis of skin cancer gene expression profiles. By comprehensively detecting the expression levels of all transcripts, it has preliminarily revealed the heterogeneity of skin cancer. These findings offer potential biomarkers for the diagnosis and prognosis of malignant skin tumors. Furthermore, it elucidates the co-evolution of tumor phenotypes during cellular malignant transformation, pointing the way toward potential targets for precision medicine. However, RNA-seq technology necessitates reverse transcription prior to sequencing, which has the potential to result in the loss of transcriptomic information (69). The scRNA-seq technology has been demonstrated to address the limitations of conventional transcriptomics, which has been shown to overlook intercellular heterogeneity and intracellular gene expression differences. This approach offers a novel perspective on the identification of novel cell subtypes, the mechanisms of tumor growth, and drug resistance mechanisms. It provides reliable evidence that can guide precision medicine. For instance, the immunosuppressive TME characteristic of MM and the heterogeneity of CAF subtypes in SCC are key factors influencing the efficacy of subsequent treatments. However, the implementation of scRNA-seq imposes elevated standards for the quality of the samples. Some skin cancer lesions are inherently small, failing to meet the sample requirements for scRNA-seq, which impedes subsequent work. The tissue dissociation process inherent to scRNA-seq is the cause of the loss of cellular spatial positioning. This consequent loss of critical information poses a fundamental challenge to deciphering spatial interactions between cells in skin cancer. ST-seq has been developed to address some of the limitations of scRNA-seq, thereby revealing the spatial distribution and functional status of tumor cells within tissues. For instance, ST-seq analysis reveals that the overall spatial distribution of cell subpopulations in invasive BCC differs fundamentally from that observed in other subtypes. The implementation of ST-seq technology is currently hindered by two significant challenges: the initial cost of the technology and the complexity of data analysis. As a cutting-edge extension of ST-seq, spatiotemporal omics technology has the capacity to analyze transcriptomic data from different time points to elucidate dynamic changes during skin tumor initiation, progression and treatment. This capability provides more comprehensive and reliable information for exploring skin tumor pathogenesis, progression, and precision therapy.

**Table 2:** Representative transcriptomic studies of skin cancer.

Cancer type	Transcriptomics technology	Targets	Key signaling pathways or drug resistance mechanisms	Reference
MM	RNA-seq	MITF, AXL	BRAF/MEK, anti-PD-1 antibody	(21)
MM	RNA-seq, scRNA-seq	MITF, SOX10, SOX9, JUN, ZEB1	IFNγ signaling pathway, PIT induction pathway, PD-L1 expression	(22)
MM	scRNA-seq, ST-seq	MITF	fatty acid oxidation pathway	(23)
MM	ST-seq	S100b^+^	Immune-modulating pathways	(27)
MM	scRNA-seq	IRF1, HLA-E, MDK	Stimulates angiogenesis through interaction with endothelial cells	(28)
MM	RNA-seq, scRNA-seq	MITF, EGFR, PDGFR, EPHA 2, AXL, NGF	the MAPK pathway	(31)
MM	RNA-seq	CCNB2, ARHGAP30, SEMA4D	the cell cycle process and the immune response process	(32)
MM	Genome, RNA-seq	PTEN, BRAF, MHC	IFNγ signaling pathway, MHC-I and MHC-II pathways	(36)
MM	RNA-seq	TNFSF9, TGFB2, LILRA3, IFIT2, EIF2AK2	TGFβ pathway, TNF pathway, B-cell activation pathway	(40)
BCC	scRNA-seq	MDK, HLA-CD8A	MHC-I pathway	(47)
BCC	RNA-seq	POSTN, WISP1	integrin and Wnt/β-catenin signaling pathways	(44)
BCC	scRNA-seq	BCAM, EPCAM, WNT5A	the HSP pathway	(43)
BCC	scRNA-seq	PTCH1/2, HHIP	the hedgehog signaling pathway	(49)
BCC	scRNA-seq	LYPD3, TACSTD2, LY6D	JNK/Jun and TGFB/Smad3 signaling pathway	(52)
BCC	scRNA-seq, ST-seq	PCYT2, ETNK1	the phosphatidylethanolamine (PE) synthesis pathway	(53)
BCC	scRNA-seq, ST-seq	IL1, OSM	NF-κB/IL1 signaling pathway	(54)
cSCC	scRNA-seq	S100A9, FABP5	NF-κB signaling pathway	(58)
cSCC	RNA-seq	SNIAL, CDH1, SLUG	TGF-β2 signaling pathway	(59)
cSCC	RNA-seq, scRNA-seq	iCAF, myCAF	CXCL12/CXCR4 pathway, p38 MAPK signaling pathway	(61)
cSCC	scRNA-seq, ST-seq	VIM, ITGA5	EMT	(64)
cSCC	scRNA-seq	TDO2	the PI3K-Akt signaling pathway	(62)
cSCC	RNA-seq	FSTL1	the THOC7-AS1/OCT1/FSTL1 axis	(65)
cSCC	RNA-seq	CCL-20, CXCL-8	recruitment of regulatory T cells, induction of Treg migration	(67)

In the study of skin malignancies, traditional RNA-seq, scRNA-seq, and ST-seq are functionally complementary. Through integrated approaches, such as WGCNA, pathway enrichment analysis, and spatial signaling networks, these technologies work synergistically to reveal molecular mechanisms at a higher resolution. Integrating human skin scRNA-seq with corresponding ST-seq data has deepened our understanding of the cellular composition of human skin and mapped the intercellular communication pathways among the skin’s major cell subpopulations (70). In the progression of cSCC, WGCNA screening based on bulk RNA-seq identified lncRNA modules associated with tumorigenesis and development. Bone et al. determined which lncRNAs are implicated in cSCC tumor progression by integrating ST-seq for localization and *in situ* validation of candidate lncRNAs (71). Additionally, the invasive “spatial DEG signature” derived from ST-seq analysis was mapped back to scRNA-seq cell subpopulations. This cross-validation confirmed key genes that were significantly associated with BCC infiltration (5). By combining RNA-seq, scRNA-seq, and ST-seq, cross-validation overcomes the limitations of individual methods. This enables the construction of a more comprehensive transcriptomic map of skin cancer. This significantly improves our understanding of skin cancer biology.

The technology’s clinical and preclinical evidence, derived from multi-center clinical sample analysis, *in vitro* and *in vivo* experimental validation, and cross-study data integration, supports its use in early skin cancer screening, drug resistance mechanism analysis, treatment efficacy prediction, and personalized therapy. This underscores its substantial translational potential in this field. In the future, with the continuous development of high-throughput sequencing technology, the joint development of multiple fields such as bioinformatics, pharmacology, and chemistry, and the mutual cooperation of omics technologies such as genomics, proteomics, and immunomics, we will be able to conduct a more comprehensive exploration of the functions and states of malignant skin tumor cells. This will provide a deeper understanding of the nature of the disease and provide a theoretical basis for personalized precision medicine.
